# Supplementary material for: Accelerating amorphous polymer electrolyte screening by learning to reduce errors in molecular dynamics simulated properties
Source: Nat Commun. 2022 Jun 14;13:3415. doi: 10.1038/s41467-022-30994-1 (PMC9197847; doi:10.1038/s41467-022-30994-1)
Supplement: Supplementary file 1 — Supplementary information [file 41467_2022_30994_MOESM1_ESM.pdf]

Supplementary Information: Accelerating the screening of amorphous polymer electrolytes by learning to reduce random and systematic errors in molecular dynamics simulated properties

T. Xie et al.

SUPPLEMENTARY NOTES

Supplementary Note 1: estimate true prediction error from noisy data

We assume there exists a deterministic function f that maps from the polymer structure $\mathcal{G}$ to its true target property. However, due to the random errors associated with the initial configuration in MD simulations, the simulated target property t has a small random error ϵ,

$$t = f(\mathcal{G}) + \epsilon, \tag{1}$$

where ϵ follows a normal distribution with zero bias $\mathcal{N}(0, \eta)$. Here, we assume that ϵ is not a function of $\mathcal{G}$, i.e. different polymers have the same random error independent of their structure. This assumption is approximately correct based on the differences in conductivity of the same polymer between two independent MD simulations in the log scale (Fig. 2).

To estimate the true prediction error of our model, we write our graph neural network model as a deterministic function g that predicts polymer property based on their structure $\mathcal{G}$,

$$y = g(\mathcal{G}). \tag{2}$$

Note that we use different labels for the predicted property y and the MD simulated property t.

Under these assumptions, the mean squared error between ML predictions and MD simulated properties, i.e. apparent prediction error, is,

$$\mathrm{MSE}(y,t) = \mathop{\mathbb{E}}_{\mathcal{G}}[(y-t)^2] = \mathop{\mathbb{E}}_{\mathcal{G}}[(y - f(\mathcal{G}) - \epsilon)^2] = \mathop{\mathbb{E}}_{\mathcal{G}}[(y - f(\mathcal{G}))^2] + \mathop{\mathbb{E}}_{\mathcal{G}}[\epsilon^2]. \tag{3}$$

Note that in the last step we use the fact that $\mathbb{E}_{\mathcal{G}}[\epsilon] = 0$.

The mean squared error between two independent MD simulations for the same polymer is,

$$\mathrm{MSE}(t_1,t_2) = \mathop{\mathbb{E}}_{\mathcal{G}}[(t_1-t_2)^2] = \mathop{\mathbb{E}}_{\mathcal{G}}[(\epsilon_1-\epsilon_2)^2] = 2\mathop{\mathbb{E}}_{\mathcal{G}}[\epsilon^2]. \tag{4}$$

Therefore, the mean squared error between ML predictions and the true target property, i.e. true prediction error, is,

$$\mathrm{MSE}(y, f(\mathcal{G})) = \mathop{\mathbb{E}}_{\mathcal{G}}[(y - f(\mathcal{G}))^2] = \mathrm{MSE}(y,t) - \mathrm{MSE}(t_1,t_2)/2. \tag{5}$$

Based on our predictions on 86 testing data, $\mathrm{MSE}(y,t) = 0.0173$ and $\mathrm{MSE}(t_1,t_2) = 0.0274$. Therefore, the true prediction error $\mathrm{MSE}(y, f(\mathcal{G})) = 0.0036$. In comparison, the

random error $\eta^2 \approx \mathbb{E}_{\mathcal{G}}[\epsilon^2] = 0.0137$. Remember that random errors can be reduced by running multiple MD simulations on the same polymers and computing the mean of target properties. Since $\eta_n = \eta/\sqrt{n}$, we estimate our ML prediction accuracy is approximately the accuracy of running $3.8 \approx 4$ MD simulations for each polymer. We note that uncertainty of this estimation is likely high due to the small size of test data and the relatively strong assumption that the random noise is Gassuian.

Supplementary Note 2: random forest model

Since our dataset is relatively small, we develop a simpler random forest (RF) model to compare its performance with our GNN model in both random and systematic error reductions. We use the Morgan fingerprint to featurize the molecular structure of the polymers and then build a RF regression model using scikit-learn to predict the properties. For the multi-task model, we use a first RF to predict the 5 ns MD properties, and then concatenate the predicted values with the Morgan fingerprint as the input features to train a second RF model. This model has a similar architecture with our multi-task GNN model but is fully composed of random forests. We also experimented a model that uses a linear model to replace the second RF, but it suffers from numerical instability so we do not report the results here.

Supplementary Note 3: reason for choosing the simulation time

We choose 5 ns as the simulation time of our short MD simulation because we need to run 5 ns MD prior to sample and relax equilibrium structure of the amorphous polymers. A shorter simulation time than 5 ns does not save total simulation time because the 5 ns MD needed for relaxation cannot be reduced. We choose 50 ns as the simulation time of our long MD simulation because we empirically find that 50 ns is enough to achieve good agreement with experiments. To apply our approach to other systems, the short and long MD simulation time should be chosen based on the specificity of the system.

SUPPLEMENTARY FIGURES

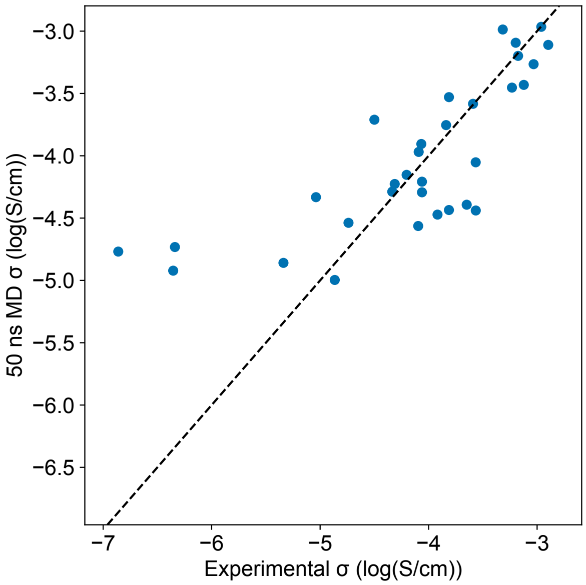

Supplementary Figure 1. Comparison between 50 ns MD simulated conductivity and experimental conductivity reported in literature at the same salt concentration and temperature.

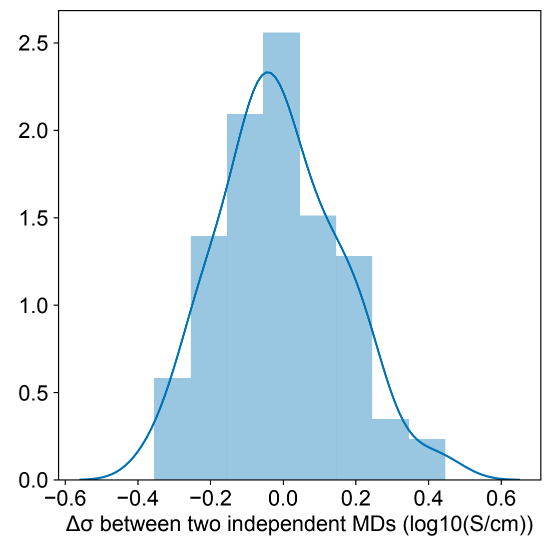

Supplementary Figure 2. Differences in conductivity of the same polymer between two independent 5 ns molecular dynamics simulations.

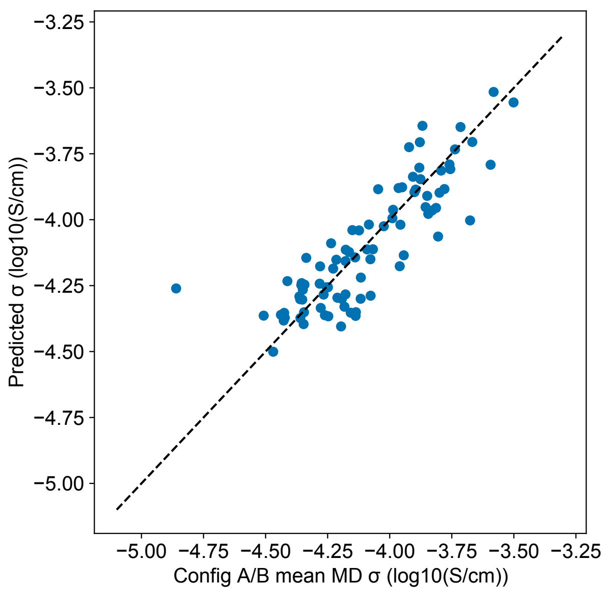

Supplementary Figure 3. Scatter plot comparing the predicted conductivity using random forest model and computed mean conductivity from two independent initializations (config A and config B) in the test dataset.

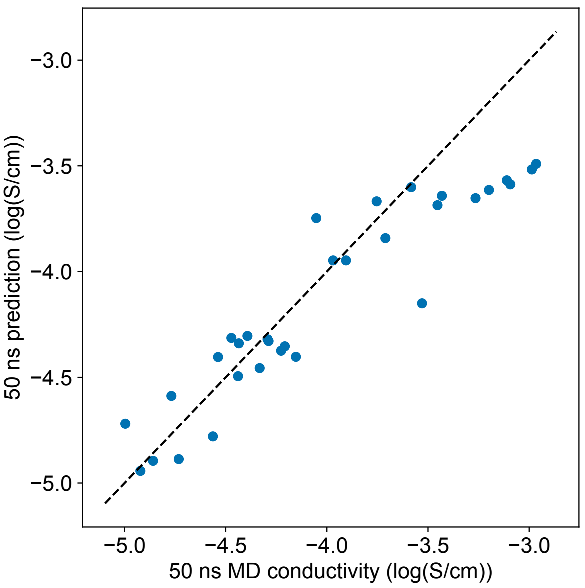

Supplementary Figure 4. Comparison between 50 ns MD and predicted conductivity for the polymers from literature.

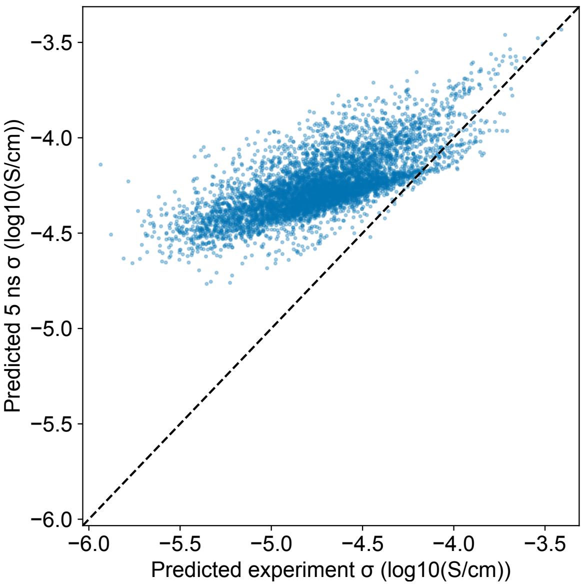

Supplementary Figure 5. Comparison between the prediction of experimental conductivities and 5 ns conductivities for 6247 polymers in the search space. The model is trained with 5 ns data and experimental data with the multi-task GCN model.

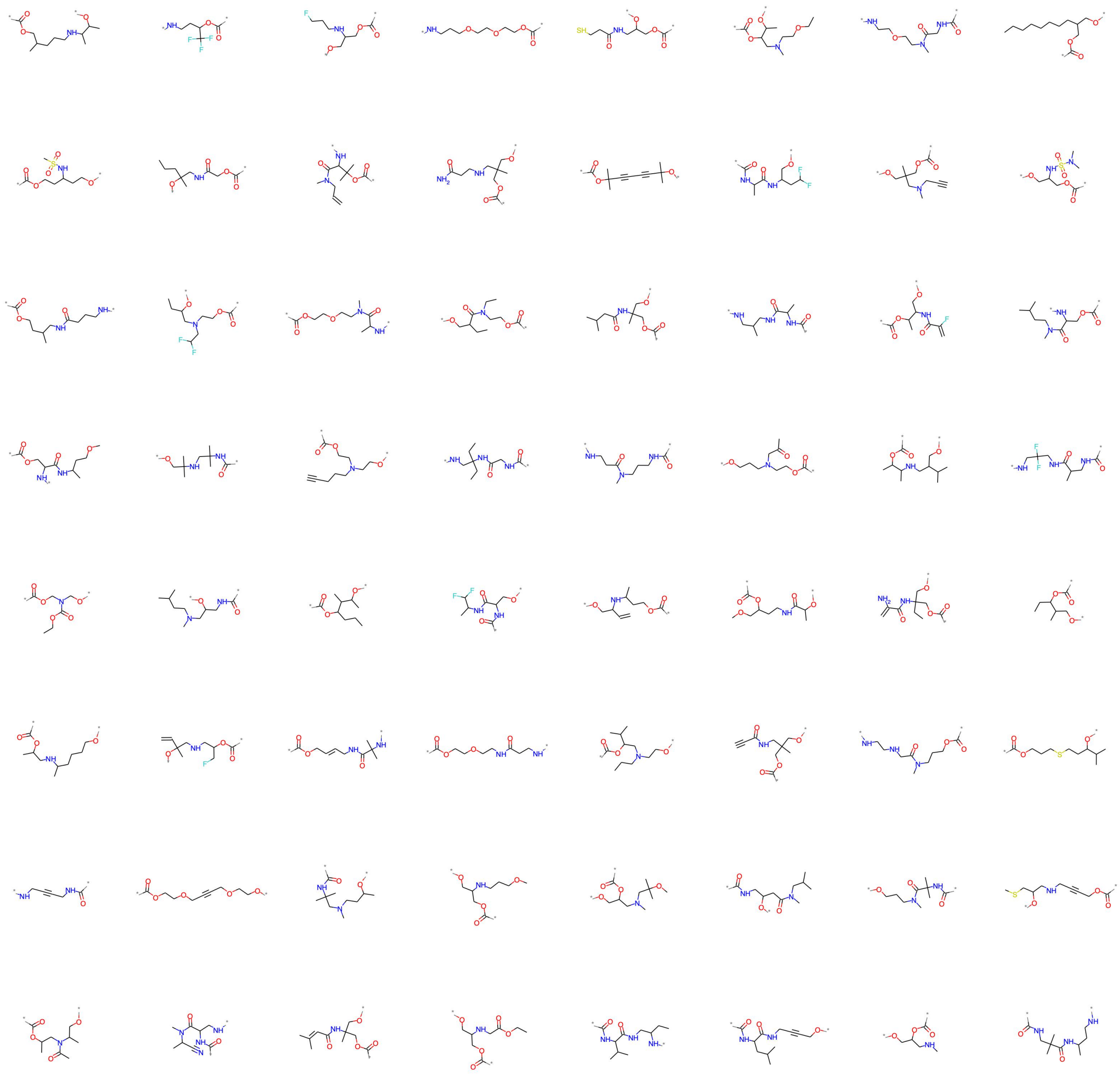

Supplementary Figure 6. Randomly sampled polymers from the 6247 search space (* denotes the connecting sites of the monomers).

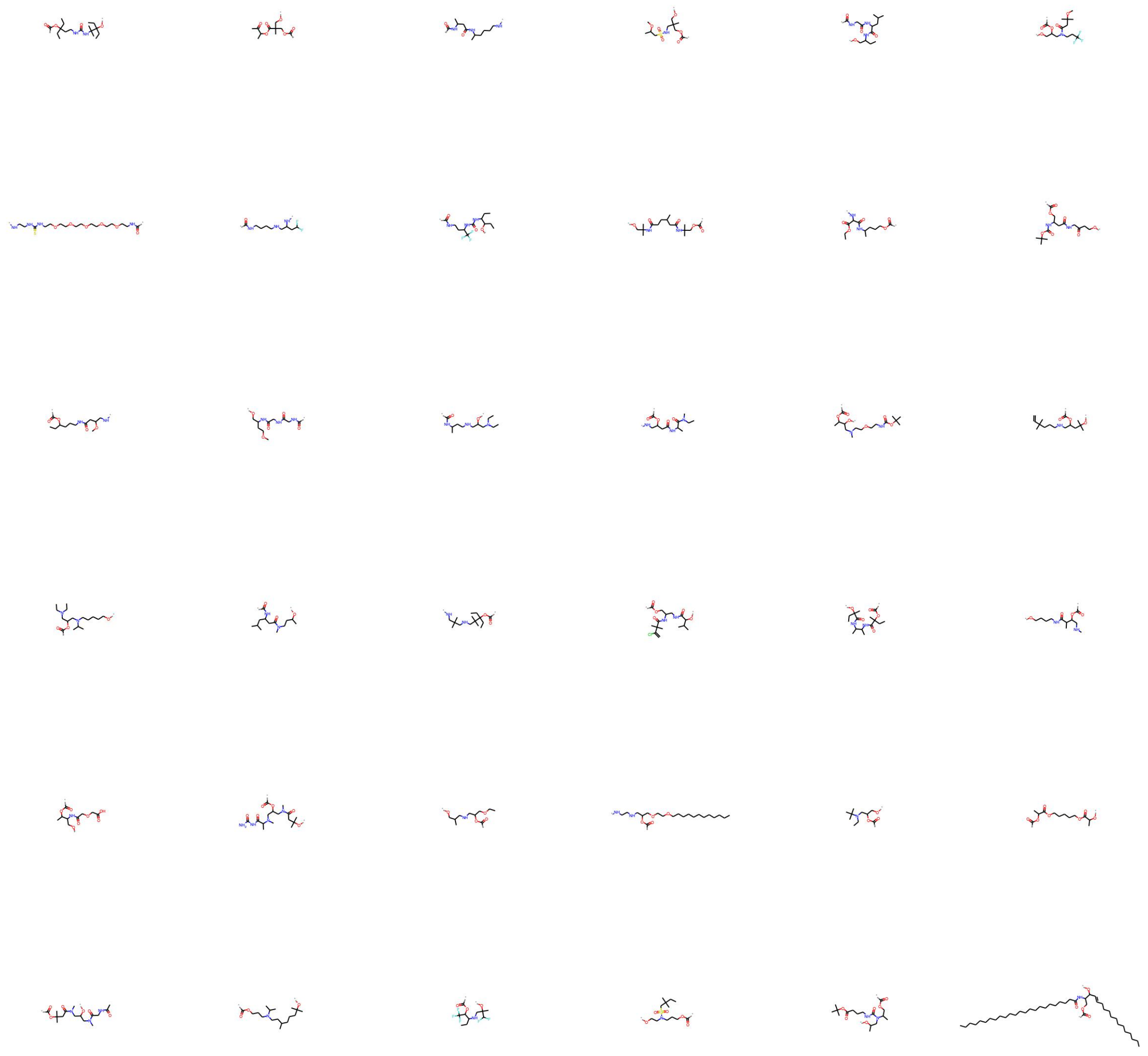

Supplementary Figure 7. Randomly sampled polymers from the 53362 candidate space (* denotes the connecting sites of the monomers).

SUPPLEMENTARY TABLES

Supplementary Table 1. Comparison of the mean absolute errors (MAEs) on predicting 50 ns MD simulated properties between different approaches. For each property, interpolation and extrapolation performance are represented by labels without and with the * symbol. Uncertainties are the standard deviations of MAEs from 10-fold cross validation (CV).

Method	σ	σ^*	D_{Li}	D^*_{Li}	D_{TFSI}	D^*_{TFSI}	D_{Poly}	D^*_{Poly}
GCN CV	0.093± 0.017	0.186± 0.053	0.106± 0.016	0.209± 0.050	0.101± 0.020	0.181± 0.028	0.072± 0.019	0.114± 0.030
RF CV	0.141± 0.008	0.305± 0.017	0.111± 0.005	0.240± 0.007	0.118± 0.012	0.274± 0.025	0.100± 0.013	0.188± 0.014

Supplementary Table 2. Molecular structure, simulated conductivity, and predicted conductivity for the top polymers in the search space. [*] denotes the connecting sites of the monomers.

SMILES	50 ns MD σ	Predicted σ
CN(CCCO[*])CCOCCOC(=O)[*]	-3.74	-3.95
O=C([*])NCCOCCOCCOCCO[*]	-3.76	-3.73
O=C([*])OCCOCCCCOCCO[*]	-3.85	-3.90
O=C([*])OCCCSCCOCCO[*]	-3.97	-3.95
O=C([*])OCCCOCCCOCCCO[*]	-4.00	-3.94
C=CCN(CCO[*])CCOCCOC(=O)[*]	-4.00	-3.95
O=C([*])NCCOCCOCCOCCN[*]	-4.00	-3.82
O=C([*])OCCNCCOCCO[*]	-4.02	-3.85
NOCCNCC(COC(=O)[*])O[*]	-4.10	-3.93
CN(CCO[*])CCOCCOC(=O)[*]	-4.19	-3.92

Supplementary Table 3. Molecular structure, simulated conductivity, and predicted conductivity for the top polymers in the candidate space. [*] denotes the connecting sites of the monomers.

SMILES	50 ns MD σ	Predicted σ
O=C([*])OCCOCCOCCOCCOCCOCCOCCOCCOCCOC COCCOCCOCCOCCOCCOCCOCCO[*]	-3.25	-3.42
O=C([*])NCCOCCOCCOCCOCCOCCOCCOCCOCCOC COCCOCCOCCOCCOCCOCCOCCOCCN[*]	-3.32	-3.48
O=C([*])NCCOCCOCCOCCOCCOCCOCCOCCOCCOC COCCO[*]	-3.32	-3.49
O=C([*])OCCOCCOCCOCCOCCOCCOCCOCCOCCOC COCCOCCOCCN[*]	-3.36	-3.47
O=C([*])OCCOCCOCCOCCOCCNCCOCCOCCOCCOCCO[*]	-3.39	-3.50
CN(CCOCCOCCOCCO[*])CCOCCOCCOCCOC(=O)[*]	-3.39	-3.54
C=CCOCC(CNCCOCCOCCOCCOCCOCCO[*])OC(=O)[*]	-3.54	-3.67
C=CCCC(CNCCOCCOCCOCCOCCOCCOC(=O)[*])O[*]	-3.56	-3.70
C=CC(CNCCOCCOCCOCCOCCOCCO[*])OC(=O)[*]	-3.59	-3.63
CC(CNCCOCCOCCOCCOCCOCCOC(=O)[*])O[*]	-3.62	-3.63
CCC(CNCCOCCOCCOCCOCCOCCOC(=O)[*])O[*]	-3.65	-3.67
CCOCC(CNCCOCCOCCOCCOCCOCCO[*])OC(=O)[*]	-3.65	-3.68
COCC(CNCCOCCOCCOCCOCCOCCO[*])OC(=O)[*]	-3.66	-3.61
CC(C)OCC(CNCCOCCOCCOCCOCCOCCO[*])OC(=O)[*]	-3.70	-3.70

Supplementary Table 4. Molecular structure, experiment molecular weight, experiment conductivity, and predicted conductivity for polymers from literature. [*] denotes the connecting sites of the monomers.

SMILES	Mn or Mw	Experiment σ	Predicted σ
[*]CCOCCOCCOCCOCCOCC[*]	6700	-2.90 [27]	-3.57
[*]OCC[*]	5000	-2.96 [4]	-3.49
[*]CCCCCCOCCOCCOCCOCCOCC[*]	19000	-3.03 [4]	-3.65
[*]CCCCOCCOCCOCCOCC[*]	4700	-3.12 [4]	-3.64
[*]CCCCOCCOCCOCCOCCOCC[*]	7100	-3.18 [4]	-3.61
[*]CCOCCOCCOCCOCC[*]	7400	-3.20 [4]	-3.59
[*]CCCCCCOCCOCCOCCOCC[*]	12900	-3.23 [4]	-3.69
[*]OCOCCOCC[*]	55000	-3.32 [4]	-3.52
[*]OC(CCCCC[*])=O	338000	-3.57 [72]	-4.49
[*]OC(=O)OCCOCCOCC[*]	35800	-3.57 [73]	-3.75
[*]OC(=O)OCCOCCOCCOCCOCCOCC[*]	21900	-3.59 [73]	-3.60
[*]OC(=O)OCCCCCCCCCCCC[*]	8100	-3.65 [6]	-4.30
[*]CC(C)O[*]	250000	-3.81 [35]	-4.15
[*]OC(=O)OCCCCCCC[*]	14800	-3.81 [6]	-4.34
[*]OC(=O)OCCOCCOCCOCC[*]	32400	-3.84 [73]	-3.67
[*]OC(=O)OCCCCCCCCCC[*]	8000	-3.92 [6]	-4.31
[*]OC(=O)OCCCCCC[*]	25100	-4.06 [6]	-4.35
[*]OC(=O)OCCCCCCCCC[*]	16100	-4.06 [6]	-4.32
[*]OC(=O)OCCOCC[*]	7603	-4.07 [34]	-3.95
[*]C(=O)CCCC(=O)OC(C)CO[*]	8800	-4.10 [4]	-4.78
[*]OC(=O)OCCCC[*]	43300	-4.20 [6]	-4.40
[*]OC(=O)OCCCCC[*]	27700	-4.31 [6]	-4.37
[*]OC(=O)OCCCCCCCC[*]	15300	-4.34 [6]	-4.33
[*]NCC[*]	10000	-4.50 [33]	-3.84
[*]C(=O)COCC(=O)OC(C)CO[*]	8000	-4.74 [4]	-4.40
[*]OC(=O)OCC(CC)(COCC=C)C[*]	10062	-4.87 [30]	-4.72
[*]OC(=O)OCCC[*]	368000	-5.04 [74]	-4.46
[*]OC(=O)OC(C)C[*]	50000	-5.34 [29]	-4.90
[*]OC(=O)OC(CC)C[*]	26000	-6.34 [29]	-4.89
[*]OC(=O)OC(CCC)C[*]	12000	-6.35 [29]	-4.94
[*]OC(=O)OCC(OC)(OC)C[*]	21000	-6.86 [32]	-4.59

Supplementary Table 5. Atom features.

Feature	Description
Atom type	Atomic number of elements (one-hot)
Degree	Atom degree (one-hot)
Formal charge	Formal charge of atoms (one-hot)
Number of hydrogen	Number of connected hydrogen atoms (one-hot)
Hybridization	Hybridization type of the atomic orbitals (one-hot)
Aromatic	Whether the atom belongs to an aromatic ring (binary)
Ring	Whether the atom belongs to a ring (binary)

Supplementary Table 6. Bond features.

Feature	Description
Bond type	Type of the bond, e.g. single, double, aromatic (one-hot)
Stereochemistry	Stereochemistry of the bond (one-hot)
Conjugated	Whether the bond is conjugated (binary)

Supplementary Table 7. Partial charges of the ions.

Species	Charge (e)
Li	+0.7000
S	+0.3395
C	+0.2100
F	−0.0826
O	−0.2513
N	−0.2982
