# Supplementary material for: Accelerating amorphous polymer electrolyte screening by learning to reduce errors in molecular dynamics simulated properties
Source: Nat Commun. 2022 Jun 14;13:3415. doi: 10.1038/s41467-022-30994-1 (PMC9197847; doi:10.1038/s41467-022-30994-1)
Supplement: Supplementary file 3 — Description of Additional Supplementary Files [file 41467_2022_30994_MOESM3_ESM.docx]

File Name: Supplementary Data 1

Description: Includes 1) LogP toy dataset, 2) 5 ns and 50 ns MD datasets, 3) polymer structures and predicted properties for 53362 candidate space and 6247 search space, 4) polymer structures and convergence curves in Fig. 1b and Fig. 1c. Details in the readme file.
